# The Role and Mechanism of Estrogen in Perimenopausal Depression

**DOI:** 10.2174/011570159X371863250327073835

**Published:** 2025-04-24

**Authors:** Yaqi Liu, Xiying Fu, Boyun Guan, Ranji Cui, Wei Yang

**Affiliations:** 1 Jilin Provincial Key Laboratory on Molecular and Chemical Genetics, The Second Hospital of Jilin University, Changchun, Jilin Province, China;; 2 Department of Neurology, The Second Hospital of Jilin University, Changchun, Jilin Province, China;; 3 Department of Endocrinology, The Second Hospital of Jilin University, Changchun, Jilin Province, China

**Keywords:** Estrogen, perimenopausal depression, estrogen receptor, HPA axis, gut microbes, brain-gut peptide

## Abstract

Depression is a severe psychiatric disorder characterized by high prevalence rates, elevated suicide risks, and significant relapse rates. Women, particularly during the perimenopausal period, are more vulnerable to developing depression. Fluctuations in estrogen levels during perimenopause can heighten a woman's sensitivity to psychosocial stress. Clinical trials have demonstrated the short-term antidepressant efficacy of estradiol in perimenopausal women. However, the precise mechanisms through which estrogen influences mood disorders during perimenopause remain unclear. This review summarizes the risk factors associated with perimenopausal depression (PMD), examines current research on estrogen therapy, and explores the potential mechanisms and related pathological processes involved in estrogen's role in treating depression. Understanding how estrogen mitigates depressive symptoms in perimenopausal women may help reduce the morbidity and mortality associated with PMD while also alleviating its socioeconomic burden.

## 1 INTRODUCTION

Depression is a prevalent and serious mental illness affecting approximately 3.8% of the global population. It is currently the leading cause of disability and death worldwide [1]. The incidence of depression is higher in females than in males, although this variation fluctuates across the lifespan [2]. The onset of depression in women is often associated with changes in serum estradiol levels during specific life stages, such as the premenstrual period (known as premenstrual anxiety disorder), the postpartum period (referred to as postpartum depression), and perimenopause [3].

Perimenopause, a transitional phase marking the shift from the reproductive stage to menopause, is characterized by significant fluctuations in ovarian hormone levels and is considered a critical period for the development of depression [4, 5]. Women in the perimenopausal phase are 2 to 3 times more likely to experience depression compared to their premenopausal counterparts [6]. The menopausal transition is associated with notable hormonal changes, particularly a significant decline in estrogen levels, which may contribute to the risk of PMD [7]. When estradiol (E_2_) levels are low, women are more vulnerable to life stress and negative emotions, thereby increasing the risk of depression [8]. A cross-sectional survey in multiple cities revealed that serum E_2_ levels were associated with depression scores in postmenopausal women in a manner that was not linearly correlated with depression severity [9]. Animal studies have shown that estrogen influences emotional behavior; for instance, ovariectomy (OVX) in rodents, which mimics estrogen deficiency, leads to symptoms of depression and anxiety, while estrogen supplementation alleviates these depression-like behaviors [10, 11]. Clinical research indicates that estradiol can effectively treat perimenopausal depression, but it is less effective for postmenopausal depression [12, 13]. This finding further suggests that fluctuations in estradiol levels, rather than absolute estrogen levels, may be a risk factor for depression in middle-aged women.

Although both human and animal studies suggest that estrogen plays a critical role in the onset and progression of PMD, the precise mechanisms underlying this relationship remain unclear [14]. The neuroendocrine mechanisms of depression, especially those influenced by the complex hormonal environment during the climacteric transition, are still poorly understood. Therefore, this narrative review aims to explore the potential relationship between estrogen fluctuations and the development of perimenopausal depression, as well as the mechanisms by which estrogen may exert its antidepressant effects.

## 2 PERIMENOPAUSAL DEPRESSION

### 2.1 The Menopause Transition Endocrinology

As a woman approaches menopause, a diminished supply of ovarian follicles initiates a cascade of endocrine changes, disrupting the hypothalamic-pituitary-ovarian axis. Although follicle-stimulating hormone (FSH) levels have been traditionally regarded as an endocrine indicator of postmenopausal status, they are less reliable for reproductive staging during the menopause transition [15, 16]. Endocrine data indicates that the reproductive staging criteria are primarily based on menstrual bleeding patterns. Due to the absence of standardized criteria for describing the stages of reproductive aging in women, the Stages of Reproductive Aging Workshop (STRAW) was established in 2001. STRAW divides menopause into reproductive, transitional, perimenopausal, and postmenopausal stages, considering the frequency of the menstrual cycle, endocrine factors, and symptoms of other organ systems [17]. Notably, the response to the menopause individual differences. Factors influencing menopause include the age at which menopausal changes occur, health status, environmental influences, and lifestyle.

During the menopause transition, E_2_ levels are also highly variable due to fluctuating FSH levels. E_2_ concentrations are particularly variable during anovulatory cycles, which occur more frequently as the transition to menopause advances. Consequently, most women who undergo a natural transition to menopause experience volatile hormonal fluctuations. This period of exposure to unstable ovarian hormone concentrations may persist for over five years [18-20].

### 2.2 Perimenopausal Depression Risk Factors

**Fig. (1):**
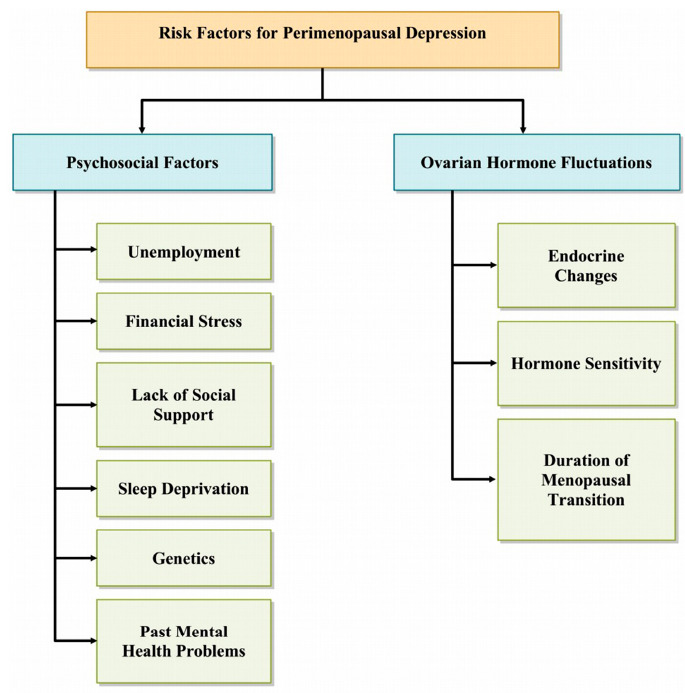
Risk factors for perimenopausal depression.

The perimenopausal period is marked by a complex interplay of social, psychological, and biological factors. Several longitudinal studies have identified key risk factors for perimenopausal depression, which can be divided into two main groups: psychosocial factors and fluctuations in ovarian hormones (Fig. **1**) [21]. Psychosocial stressors, such as unemployment, financial pressure, lack of social support, and stressful life events close to the menopause transition, are associated with an increased likelihood of experiencing depressive symptoms. Furthermore, inadequate sleep during menopause has been linked to a higher risk of PMD [22-24]. The increasing prevalence of depression among women is also thought to be influenced by genetic predispositions, previous mental health issues, and experiences of sexual abuse [25].

The recurring endocrinological changes that occur in women during adolescence, pregnancy, postpartum, and menopause may contribute to their higher risk of depression than males from puberty to old age [25]. Postpartum depression and premenstrual dysphoric disorder are strongly predictive of PMD [23]. Studies have revealed that women are more prone to develop depressive symptoms during perimenopause than at other times in their lives, even if they have no prior history of depression [23, 26]. The idea is that the altering hormonal milieu of menopause develops depressive symptoms, which contributes to the increased risk for perimenopausal depression. This is supported by the observation that women with a history of perimenopausal depression exhibit a rapid increase in depressive symptoms following an abrupt experimental withdrawal from estradiol, which is not seen in women without such a history [13]. Moreover, among women of similar age with no lifetime history of depression, those who enter the menopause transition earlier are at a higher risk of experiencing a first episode of depression [26]. PMD-vulnerable women demonstrate a higher “hormonal sensitivity” to the endocrine characteristics of the menopause transition [27]. The duration of the menopausal transition is positively related to PMD risk [28]. Interestingly, some women may have a specific reproductive phenotype that makes them more susceptible to depression [25].

## 3 ESTROGEN THERAPY

Estrogen refers to a class of steroid hormones primarily produced by the placenta and ovaries, with minor contributions from the adrenal cortex. In the human body, there are three types of estrogen: estrone, E_2_, and estriol. E_2_ has more bioactivity under normal conditions [29]. Estrogen regulates neuronal survival, proliferation, and plasticity, with neurotrophic and neuroprotective impacts on regions such as the hippocampus, cortex, amygdala, and basal forebrain, according to clinical and rodent studies [30, 31]. It also exhibits antidepressant effects by promoting neuronal growth, enhancing monoaminergic activity, and regulating the hypothalamic-pituitary-adrenal (HPA) axis [32]. Traditional antidepressants are typically used as first-line treatment for perimenopausal depression, but as the beneficial effects of estrogen on the central nervous system become more well-understood, estrogen therapy may play a role in managing perimenopausal depression.

Clinical studies have confirmed that estrogen therapy (ET) can effectively improve depression symptoms in postpartum, perimenopausal, and postmenopausal women [33-35]. Three small randomized controlled trials tested transdermal estradiol for PMD. Two had remission rates of 68 and 80%, compared to 20% and 22% in placebo groups [12, 34]. The third trial of depressed peri-and postmenopausal women without menstrual irregularity found no mood differences between transdermal estradiol, the hypnotic zolpidem, and placebo [36]. Gordon *et al*. investigated the effectiveness of transdermal estradiol plus intermittent micronized progesterone (TE+IMP) in avoiding the emergence of depressive symptoms in euthymic perimenopausal and early postmenopausal women. Twelve months of TE+IMP was found to be more efficacious than placebo in avoiding the development of clinically significant depression symptoms in originally euthymic perimenopausal and early postmenopausal women [13]. Other studies have demonstrated that combination therapy with estrogen and conventional antidepressants is more effective than using either antidepressants or estrogen alone in treating depression during menopause [37, 38]. Nagata *et al*. compared sole ET with a combination of estrogen and fluvoxamine (50 mg daily) [37]. After eight weeks, those receiving the combination therapy exhibited significantly greater improvements in depressive symptoms [37]. Similarly, Schneider *et al*. conducted a double-blind study in which participants treated with estrogen therapy plus fluoxetine (20 mg daily) showed significantly better depression ratings compared to those receiving fluoxetine alone [38]. These studies may suggest that estrogen plays a role in treating perimenopausal depression. However, long-term estrogen use is associated with certain side effects, including an increased risk of breast cancer and cardiovascular disease [39]. Therefore, evaluating the tolerability and long-term side effects of estrogen therapy is essential. Although several studies have investigated the safety of estrogen replacement therapy, most have primarily focused on its effects on bone mineral density and cardiovascular health, with insufficient attention given to its long-term safety in the treatment of depression. Consequently, the risk-benefit ratio of estrogen replacement therapy for long-term perimenopausal depression requires further investigation. For instance, strategies such as low-dose estrogen or local estrogen delivery systems could help reduce systemic side effects and should be explored. Additionally, assessing patients’ tolerance to estrogen at the initiation of treatment to avoid overtreatment is a key area for future research.

## 4 ESTROGEN'S ANTIDEPRESSANT MECHANISMS

### 4.1 Estrogen Receptor

Estrogen exerts its biological effects by binding to specific receptors. Estrogen receptors (ERs) include the classical ERs, ERα and ERβ, the non-classical ERs, the G protein-coupled receptor, and the Gq-coupled membrane estrogen receptor. ERα and ERβ are localized in the hippocampus and amygdala [40]. Recent studies have highlighted that estrogen not only regulates reproductive functions but also plays a critical role in maintaining normal brain function. Estrogen signaling pathways are implicated in the pathophysiology of depression [41]. ET exerts antidepressant effects through ERα and ERβ in the brain [42]. For instance, rats with natural estrogen receptors exhibit increased anxiety and depressive behaviors after receiving the ER blocker ICI 182,780 in the hippocampus [43].

In clinical and preclinical studies, it has been shown that estrogen fluctuation or depletion can change how serotonin works in women with depression [44-46]. The interaction between the serotonergic system and estrogen receptor activation, particularly ERβ, has been linked to the role of estrogens in mood regulation. The antidepressant-like effect of estrogen depends on the involvement of the 5-hydroxytryptamine (5-HT)1A receptor [47]. Serotonin neurons show high expression of ERβ, which is involved in their functional maintenance [48]. Furthermore, ERβ activation enhances the expression of the rate-limiting enzymes of serotonin synthesis: the tryptophan hydroxylase 1 (TPH1) and TPH2 enzymes in the dorsal raphe nucleus (DRN) [49]. Previous research has shown that ER is robustly expressed in the DRN of rodents, whereas ERα is only weakly expressed in the DRN of these species [40, 50]. Yang *et al*. demonstrated that an ERβ-selective agonist, but not an ERα-selective agonist, reduced depressive behavior in OVX rats [51]. ERβ agonists decrease the rats' passive floating and immobility behavior during the forced swimming test in the Flinders-sensitive strain, a strain selectively bred for depressive-like behaviors [52]. In ovariectomized ERβ^−/−^ and wild-type mice, the number of serotonergic neurons is drastically reduced [53]. The selective ERβ agonist prevented serotonergic neuronal changes in OVX mice [48]. Moreover, mice with permanent ERβ knockouts have more anxiety and depression, which is ineffective with E_2_ administration [53]. Moreover, the regulation of brain-derived neurotrophic factor (BDNF) by estrogen may occur through the regulation of BDNF gene expression by ERβ as an estrogen response element [54]. These findings indicate that ERβ could be a promising target for the treatment of PMD. However, several studies have found that ERT improves depression-like behaviors in OVX rats *via* ERα but not ERβ [55, 56]. ERα and corticotropin-releasing hormone (CRH) neurons share approximately 40% structural similarity, and the CRH gene promoter region has estrogen response elements. These results implied that ERα and CRH neurons might jointly regulate CRH levels by estrogen, which in turn affects the activity and response state of the HPA axis [57]. Polymorphisms in the ERα gene, such as rs9340799 and rs2234693, are associated with situational memory, mood regulation, and anxiety-related depression in older women. Moreover, the ERα gene rs9340799 polymorphism may influence the development and regression of depression [58]. The understanding of how estrogen receptors, particularly ERβ, influence mood, and serotonin function opens up possibilities for novel antidepressant strategies. Instead of relying on generalized hormonal replacement therapies, which carry a range of side effects and risks, the development of selective estrogen receptor modulators or ERβ-specific agonists could offer a more targeted approach.

### 4.2 Neurotransmitters

The “neurotransmitter hypothesis” has become a key framework for understanding the pathogenesis of depression. Research has demonstrated that estradiol modulates the serotonergic, noradrenergic, dopaminergic, and cholinergic systems in various ways [59]. The effects of estrogen on neurotransmitter systems can be summarized as follows: E_2_ increases 5-HT synthesis in the DRN, the main regulatory area of the serotonergic system in the brain [60]. 5-HT, through its receptors, exerts mood-regulating effects, with activation of the 5-HT1A receptor having antidepressant properties [47]. Gender differences exist in the regulation of 5-HT. Women are more prone to depression during premenopause, perimenopause, and postmenopause, and 5-HT responsiveness decreases during this stage. E_2_ treatment has been shown to restore 5-HT responsiveness [61]. TPH2 is a rate-limiting enzyme for serotonin synthesis, while monoamine oxidase A (MAO-A) is a key enzyme responsible for serotonin degradation in the brain [62, 63]. Both of these enzymes are potential targets for estrogen's antidepressant effects. Rekkas *et al*. conducted a study using carbon 11–labelled harmine positron emission tomography to examine the effects of menopause and fluctuating estradiol levels on MAOA binding [64]. This study found that estradiol regulates 5-HT synthesis, increases 5-H2A receptor binding, and interferes with extracellular 5-HT clearance by regulating TPH2 gene expression. Additionally, estradiol reduced serotonin catabolism by lowering MAO-A and RNA expression and enzyme activity. E_2_ treatment increased TPH2 mRNA content in particular subregions of the DRN in OVX rats [65] and decreased MAO-A expression in the dorsal spine of OVX macaques [66], both of which are essential for alleviating depression-like behavior. In the dopaminergic system, estrogen treatment increased tyrosine hydroxylase mRNA [67] and enhanced dopaminergic neuronal activity [68]. Chronic E_2_ treatment increased dopamine (DA) D2 receptor density in the striatum and nucleus accumbens, while D2 mRNA levels remained unchanged in the striatum, suggesting that E_2_ regulates D2 receptor density through non-genomic effects [69]. Becker *et al*. found a significant decrease in striatal DA concentrations in OVX rats. Exogenous E_2_ supplementation increased DA concentrations and promoted DA release in the hypothalamus and the anterior pituitary, thereby improving OVX-induced depression-like behaviors [70]. Furthermore, a rise in estrogen levels in rats during proestrus may increase the turnover rates of norepinephrine and dopamine [71]. E_2_ also stimulates gene expression of norepinephrine biosynthetic enzymes in rat locus coeruleus [72].

Estrogen also has an impact on depression by regulating cognitive function *via* its interaction with the cholinergic system. In ovariectomized animals, estrogen replacement mitigates the negative effects of cholinergic antagonists on spatial learning and memory. In postmenopausal women, estrogen substitutes protect against cholinergic antagonism-induced impairments in verbal working memory, learning, and attention [73-75]. Existing studies have typically focused on a single neurotransmitter system as the primary object of study, ignoring the interactions between multiple systems. For example, estrogen's regulation of the DA system may not be limited to increasing DA synthesis but may also enhance the response of mood and reward mechanisms by affecting the function of the 5-HT system. Therefore, synergistic effects of different neurotransmitter systems in perimenopausal depression should be revealed in the future through more detailed neural network studies.

### 4.3 Brain-derived Neurotrophic Factor

BDNF is a neurotrophic factor that has been extensively explored in relation to depression. In animal models of depression, acute BDNF injections into the hippocampus or lateral ventricles generate antidepressant-like effects [76, 77]. Chronic antidepressant treatment elevated BDNF levels in the cortex and hippocampus of rats, implying that BDNF upregulation is one of the mechanisms underlying the action of antidepressant drugs [78]. In OVX rats, E_2_ administration improves BDNF mRNA and protein expression in the hippocampus [79, 80]. Additionally, BDNF mRNA and protein levels fluctuate during the estrous cycle in female rats, with the highest levels occurring in late diestrus when estrogen is at its peak [81, 82].

Tyrosine receptor kinase B (TrkB), a member of the receptor tyrosine kinase family, and p75, which binds neurotrophins, are the two transmembrane receptors that interact with BDNF. Estrogen regulates two receptors in different ways [83, 84]. Through TrkB, BDNF activates numerous intracellular signaling pathways, including mitogen-activated protein kinases (MAPK), phosphatidylinositol 3-kinase (PI3K), and phospholipase C (PLC)- mediated signaling pathways, thus influencing the development and function of the nervous system [85]. These pathways are also a hallmark of rapid E_2_ signaling initiation, and E_2_ treatment is capable of triggering PI3K/Akt signaling in the hippocampus [86, 87]. The findings showed that exogenous E_2_ treatment increased the expression of phosphorylated TrkB and regulated TrkB activity in the hippocampus [88]. Estrogen receptors are colocalized with neurotrophin-sensitive neurons in the basal forebrain [89]. Estrogen promotes neurotrophin and their tyrosine kinase receptors' expression in the cerebral cortex, olfactory bulb, and hippocampus [90, 91]. The BDNF gene contains sequences that are similar to estrogen response elements found in estrogen genes [54]. cAMP response element-binding protein (CREB), a transcription factor within cells, partially regulates BDNF expression [92]. Dysregulation of CREB activity has been associated with mood disorders, such as depression [93]. The signaling of CREB and BDNF-TrkB forms a positive feedback loop, where TrkB signaling phosphorylates CREB, which in turn activates BDNF to further enhance TrkB signaling [92]. In a series of studies, E_2_ modulates BDNF expression by activating CREB through protein kinases, including MAPK, calmodulin-dependent protein kinase IV (CaMK IV), and protein kinase A. In the amygdala of ovariectomized rats, E_2_ treatment elevated the protein levels of CaMK IV, CREB, and pCREB [94]. The action mechanism of BDNF extends beyond a single receptor and signaling pathway. The previous discussion primarily focused on BDNF's regulatory effects through pathways such as TrkB and CREB. However, its interactions with other neurotransmitter systems, including 5-HT, DA, and γ-aminobutyric acid (GABA), remain underexplored. Additionally, BDNF plays a role in other brain regions, such as the prefrontal cortex and basal ganglia, where neural networks are critical for emotional regulation. Therefore, future research should place greater emphasis on the synergistic interactions between BDNF and other neurobiological mechanisms to further elucidate its complex role in perimenopausal depression.

### 4.4 Hypothalamic-pituitary-adrenal Axis

The HPA axis is a crucial neuroendocrine system component that is involved in acute and long-term stress responses and plays an essential role in the pathophysiology of depression [95]. In depression and chronic stress, the HPA axis becomes hyperactive, with a reduction in glucocorticoid receptor (GR) expression in the hypothalamus and pituitary, leading to desensitization of negative feedback regulation. This results in a continuous increase in glucocorticoid (GC) secretion, adrenal hypertrophy, and cortisol secretion [96]. E_2_ upregulates GR *via* the HPA axis and reduces plasma cortisol levels, thereby alleviating anxiety and depression-like behavior [97]. Alterations in HPA axis function may contribute significantly to the pathogenesis of female depression. Cortisol demonstrates gender differences in its response to stress and varies with the menstrual cycle and pregnancy. Throughout acute psychosocial stress, women show more pronounced negative emotional responses and reduced hippocampal activity during the low estrogen phase of the menstrual cycle [8].

**Fig. (2):**
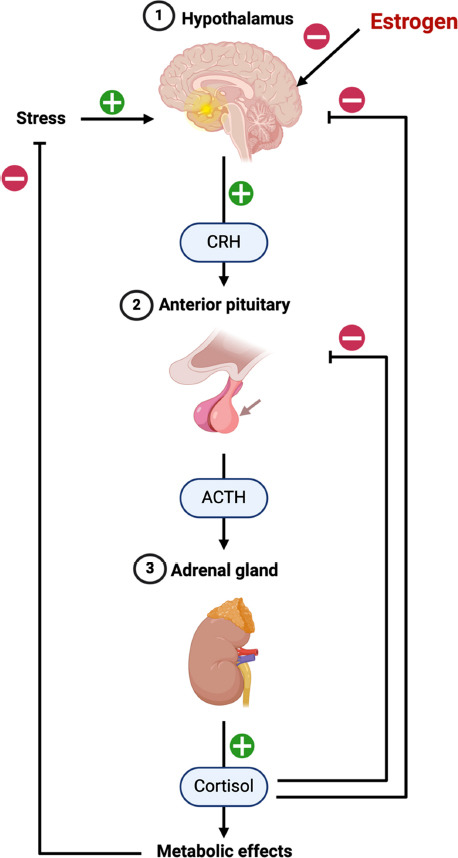
Relationship between estrogen and Hypothalamic-pituitary-adrenal axis. **Abbreviations**: CRH, corticotropin-releasing hormone; ACTH, adrenocorticotrophic hormone.

The hypothalamic-pituitary-gonadal axis interacts bidirectionally with the HPA axis [98]. According to research, chronic stress exposure exacerbates HPA axis negative feedback dysfunction in rats following long-term ovarian hormone deprivation [99]. The harmful feedback impairment is more pronounced in postmenopausal women. In a study of 36 postmenopausal women, stress was induced by giving them psychological tasks or a cold pressure test. The participants were then randomly assigned to receive either a placebo or six weeks of transdermal estradiol treatment. Only the placebo group saw significant increases in their serum levels of adrenocorticotrophic hormone (ACTH), cortisol, and neuroexcitatory hormone (NE) compared to their baseline measurements [100]. Consistent with this finding, ovariectomized monkeys who were given estradiol and/or progesterone for one month reduced the amount of CRH mRNA and protein in the paraventricular nucleus (PVN) [101]. Estradiol regulates basal and stress-induced ACTH and cortisol levels through the GC receptor and CRH [102]. In stressed rats, ERβ selective agonist significantly decreases the levels of corticosterone and ACTH [103]. ER agonists lower levels of stress hormones by acting on neuronal populations within the PVN of the hypothalamus, with oxytocin playing a key role in this process [104]. As a consequence of this, several researchers believe that estrogen supplementation therapy might be able to assist in preventing the potential for HPA axis hyperactivity during menopause by lowering the ACTH and cortisol response to CRH [105]. These findings suggest that E_2_ levels may modulate the HPA axis in women. Therefore, HPA axis dysfunction resulting from reduced E_2_ levels may contribute to the increased risk of perimenopausal depression in women (Fig. **2**).

### 4.5 Inflammatory

An increasing body of evidence suggests a strong connection between inflammation and depression. Overexpression of pro-inflammatory cytokines in the brain promotes anxiety and depression-like behaviors [106]. Patients with major depressive disorder (MDD) exhibit elevated levels of pro-inflammatory cytokines, such as interleukin-1β (IL-1β), interleukin-6 (IL-6), and tumour necrosis factor-α (TNF-α) in both their peripheral circulation and certain brain regions [107, 108]. Estrogen deficiency during menopause exacerbates immunological diseases, with perimenopausal women experiencing more frequent inflammatory responses and autoimmune disorders. Estrogen regulates the immune system by inhibiting inflammation [109]. Studies have shown that estrogen deprivation increases IL-6 production in postmenopausal women, whereas women receiving hormone replacement therapy exhibit lower serum IL-6 levels [110, 111]. Estrogen also modulates inflammatory responses of microglia and astrocytes, as well as the synthesis and release of inflammatory factors [112, 113].

**Fig. (3):**
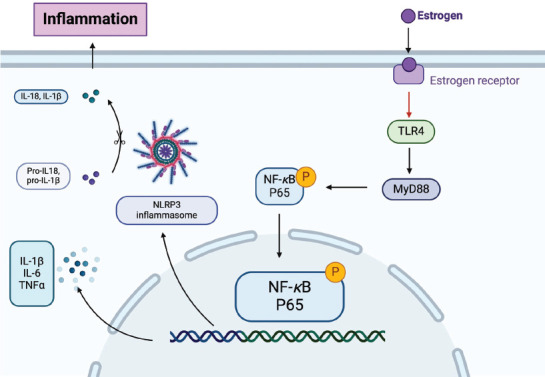
Relationship between estrogen and neuroinflammation. **Abbreviations**: TLR4, Toll-like receptor 4; MyD88, myeloid differentiation factor 88; NF-κB, nuclear factor κB; TNF-α, Tumor necrosis factor-α; IL, Interleukin; NLRP3: nucleotide-binding and oligomerization domain-like receptor family pyrin domain-containing 3.

Toll-like receptor 4 (TLR-4) and nuclear factor *κ*B (NF-*κ*B) are key molecules that activate microglia and astrocytes, and estrogen treatment prevents their expression [114-116]. E_2_, at the pregnancy level, inhibits Lipopolysaccharide (LPS)-induced DNA binding and transcriptional activity of the NF-*κ*B subunit p65 in mouse monocytic cells by blocking the subunit's nuclear translocation [117]. Similarly, the LPS-induced activation of NF-*κ*B in primary rat astrocytes is inhibited by pretreatment with high doses of E_2_ at a concentration of 10^6^ M [118]. In lymphocytic HeLa cells, elevated E_2_ levels (at pregnancy levels) significantly inhibit the phorbol-12-myristate-13-acetate-induced activation of NF-*κ*B and the degradation of inhibitory NF-*κ*B protein [119]. In rat vascular smooth muscle cells, E_2_ at pregnancy levels inhibits both constitutive and IL-1-stimulated NF-*κ*B activation. E_2_ also inhibits NF-*κ*B -mediated luciferase reporter activity and IL-6 secretion in human aorta endothelial cells at concentrations ranging from 10^10^ to 10^7^M [120]. Recently, the inflammasome nucleotide-binding and oligomerization domain-like receptor family pyrin domain-containing 3 (NLRP3), a key player in inflammatory diseases, has been linked to stress and depression [121]. NLRP3 inflammasome levels in peripheral blood mononuclear cells are elevated in patients with major depressive disorder [122]. In rodent brains, depression induced by LPS or chronic unpredictable mild stress (CUMS) is associated with NLRP3 inflammasome activation. Xu *et al*. demonstrated that estrogen deficiency led to NLRP3 inflammasome activation, and inhibiting NLRP3 inflammasomes improved OVX-induced depression-like behaviors [123]. Moreover, estrogen regulation of hippocampal inflammation and depression-like behavior is dependent on ERβ. This supports the theory that estrogen improves perimenopausal depression through anti-inflammatory actions (Fig. **3**). Although the modulation of TLR-4, NF-κB, and NLRP3 inflammasomes by estrogen is well-documented, the molecular pathways through which estrogen influences these processes remain poorly understood, particularly in the context of stress-induced neuroinflammation. Furthermore, clinical research is needed to explore combination therapies involving anti-inflammatory drugs and hormone therapy to address both immune dysfunction and hormonal imbalances underlying depression in postmenopausal women. Such therapies may include nonsteroidal anti-inflammatory drugs, cytokine inhibitors, or cannabinoid-based treatments in combination with hormone replacement therapy.

### 4.6 Synaptic Plasticity

Estrogen has long been associated with cognitive function [124]. It influences dendritic spine production and the formation of new neuronal connections, which in turn affect hippocampal neuroplasticity in women [124, 125]. The number, size, and shape of dendritic spines are critical for synaptic plasticity. Dysregulation of this process may contribute to the pathophysiology of depression [126]. The density of dendritic spines fluctuates throughout the estrous cycle and is positively correlated with varying levels of estradiol in the serum. For example, in rodents, dendritic spine density varies across the menstrual cycle, with peak density observed when E_2_ is at its highest [127]. Low estradiol levels are associated with reduced synapse density, while high estradiol levels are linked to increased synapse density. These synaptic alterations occur rapidly, with a 32% reduction in hippocampal synapse density occurring within approximately 24 hours between the proestrus and estrus stages of the estrous cycle [128]. Additionally, *in vivo*, comparisons of synapse density between male and female mice revealed a marked sex difference: the density of synapses in males was comparable to that of ovariectomized females, but it was twice as high in intact cyclic females [129].

OVX leads to the loss of dendritic spines, which is rescued by estradiol treatment, suggesting that ovarian-derived estradiol induces dendritic spine formation in the hippocampus [125]. Research has shown that the hippocampus CA1 region is most affected by estrogen-induced synaptogenesis [127, 128]. Recent research suggests that hippocampal-derived estradiol, rather than ovarian estradiol, has a role in synaptogenesis in the female hippocampus [124]. In females, the hypothalamus releases gonadotropin-releasing hormone to stimulate neurosteroid synthesis, which likely underlies the estrous cycle-related changes in spine synapse density in the hippocampus. Consequently, the amount of estradiol synthesized in the hippocampus is influenced by peripheral serum estradiol concentrations [125]. Several studies have demonstrated that estrogen receptor β mediates estrogen’s effects on hippocampus synaptic plasticity and memory and that selective ERβ agonists enhance hippocampal synaptic protein levels [130, 131]. However, the precise mechanisms by which ERβ regulates estrogen’s effects on neuroplasticity and memory remain unclear. Is ERβ primarily responsible for estrogen’s neuroprotective effects in the hippocampus, or do both ERα and ERβ contribute differently under various conditions? Further studies on receptor-specific agonists could provide insight into how to selectively target estrogen’s beneficial effects on cognition while minimizing the risks associated with systemic estrogen therapy. Although human studies have not conclusively supported estrogen's role in menopause-related cognitive impairment, studies in nonhuman primates have revealed a clear role for estrogen in cognition. In old nonhuman primates, for example, ovariectomy induces spatial memory deficits that can be restored with cyclic, low-dose estrogen administration [132]. Therefore, the effects of decreased estradiol levels on neuroplasticity may explain why women are more vulnerable to depressive disorders during the perimenopausal stage.

### 4.7 Neurogenesis

Estrogen is a crucial hormone in maintaining female physiological functions, and its role in the brain, particularly in the regulation of neurogenesis, may contribute significantly to the development of perimenopausal depression [133, 134]. Neurogenesis refers to the process of generating new neurons in the adult brain, especially in the hippocampus, a region crucial for emotional regulation, memory, and learning, and plays a pivotal role in the pathogenesis of depression [135]. Chen *et al*. suggested that the increased incidence of depression in perimenopausal women may be partially attributed to diminished estrogen's protective effects on the nervous system, particularly the decline in neurogenic capacity in key emotional regulation regions such as the hippocampus [136]. Estrogen can enhance neurogenesis in the adult hippocampus in a time- and dose-dependent manner, primarily by increasing neuron numbers through cell proliferation [137]. For example, in OVX mice, serum estradiol levels rapidly decrease within 24 hours, accompanied by a significant reduction in cell proliferation and the generation of immature neurons [138]. This phenomenon can be reversed with acute estrogen exposure. In adult rats, estradiol and estrone exposure for 30 minutes to 2 hours within 7 days after ovariectomy increases the rate of cell proliferation, whereas estradiol benzoate exposure for 4 hours results in a slight increase [138]. BDNF, a key regulator of neurogenesis, neuronal growth, and synaptic plasticity, is closely linked to depression; low BDNF levels are associated with depressive symptoms [139]. Estrogen promotes neuronal growth and repair by upregulating BDNF expression, thereby improving mood and cognitive function [140]. Furthermore, estrogen can rapidly activate signaling pathways that lead to the translation and modification of synaptic proteins, actin remodeling, and the formation of dendritic spines and synapses, thereby enhancing neurogenesis through neuronal activity regulation [141, 142]. For instance, estradiol can rapidly induce calcium influx *via* L-type calcium channels, increase N-methyl-D-aspartate receptor-mediated excitability, and reduce GABA-mediated inhibition [143]. Evidence suggests that locally synthesized estrogen in the hippocampus may play a crucial role in restoring cell proliferation after long-term estrogen deprivation. For example, after long-term ovariectomy, locally synthesized estrogen can restart cell proliferation [142], which may explain why high-dose estrogen treatment has limited effects on cell proliferation after prolonged estrogen deprivation.

The effects of estrogen on synaptic plasticity and neurogenesis appear to be time-sensitive, with the most beneficial outcomes occurring when estrogen levels are elevated, such as during the follicular phase of the menstrual cycle or hormone replacement therapy, or in the early stages of perimenopause. The timing hypothesis of perimenopausal cognitive decline suggests that early estrogen intervention may prevent or alleviate the most severe cognitive impairments, whereas delayed intervention may be less effective or even counterproductive. Identifying the optimal “window of opportunity” for estrogen treatment in menopause-related cognitive dysfunction and depression remains a critical area for future research.

### 4.8 Oxidative Stress

Oxidative stress refers to a physiological state characterized by an excess accumulation of reactive oxygen species (ROS) and free radicals, which overwhelms the capacity of the antioxidant system to neutralize them, resulting in cellular damage [144]. The relationship between estrogen and oxidative stress has gained increasing attention in the pathogenesis of perimenopausal depression [145]. Estrogen is considered a natural antioxidant that reduces ROS generation and protects cells from oxidative damage by activating various antioxidant enzyme systems, including superoxide dismutase, glutathione peroxidase, and catalase. These enzymes are integral components of the body's antioxidant defense system, capable of scavenging free radicals and preventing cellular oxidative damage [146, 147]. Studies have demonstrated that estrogen reduces oxidative stress in mouse models by upregulating the expression of superoxide dismutase (SOD2), thereby mitigating the onset of neurodegenerative diseases [148-150]. Additionally, estrogen's protective antioxidant effects on the cerebral cortex and hippocampus have been confirmed. Estrogen not only directly affects antioxidant enzymes but also regulates the expression of antioxidant genes through the activation of estrogen receptors [150, 151]. Specifically, estrogen modulates the expression of antioxidant genes, such as SOD2, *via* ERα, reducing ROS levels and enhancing cellular resistance to oxidative stress [152, 153]. Furthermore, ERβ has been found to play a key role in regulating the oxidative stress response [148, 154]. Lipid peroxidation, a common manifestation of oxidative stress, occurs when excess ROS reacts with fatty acids in the cell membrane, leading to lipid peroxidation, which triggers cellular damage and inflammatory responses [155]. Estrogen can inhibit oxidative stress-induced cell damage by directly reducing lipid peroxidation [147]. Moreover, estrogen helps cells maintain stability during oxidative stress by inducing the synthesis of molecules such as heat shock proteins. These proteins play protective roles by repairing damage and restoring cellular function. By increasing the expression of these proteins, estrogen enhances cellular tolerance to oxidative stress [156]. Although estrogen exhibits significant antioxidant effects, in certain circumstances, estrogen metabolites may exacerbate oxidative stress, particularly when estrogen levels are excessively high or estrogen metabolism is abnormal [147].

The interaction between estrogen and oxidative stress has been widely studied in clinical research. Estrogen replacement therapy is commonly used to alleviate perimenopausal symptoms, but its impact on oxidative stress remains controversial. One study on estrogen replacement therapy indicated that estrogen treatment significantly reduces oxidative stress markers in the blood, such as 8-hydroxy-2'-deoxyguanosine and malondialdehyde, while improving depressive symptoms [157]. However, another study suggested that high doses of estrogen may increase oxidative stress, leading to redox imbalance, which could exacerbate depressive symptoms or other health issues [158]. The differences between these studies may arise from several factors: (1) Variations in study design, such as population, treatment dosage, and treatment duration, could contribute to inconsistent results. (2) Genetic differences, lifestyle factors, and underlying health conditions may significantly influence treatment outcomes. Individual responses to estrogen can lead to markedly different effects, which may explain why some patients do not experience the expected antioxidant benefits or even develop side effects from estrogen replacement therapy. (3) There may be a “U-shaped” relationship between estrogen dosage and its effects. In some cases, moderate estrogen levels exhibit antioxidant effects, while excessively high doses may induce more oxidative damage. This dose-dependent relationship has not yet been fully explored or verified. Therefore, future research should focus on optimizing dosage, personalizing treatment, evaluating the role of metabolic products, and conducting comprehensive evaluations of multiple biomarkers to better guide clinical treatment.

### 4.9 Energy Metabolism and Mitochondrial Activity

The critical role of E_2_ in regulating insulin sensitivity and energy expenditure establishes a key link between hormonal fluctuations and metabolic health. Perimenopause is a critical period when women are at increased risk of developing metabolic syndrome, including insulin resistance, obesity, and hyperglycemia [159, 160]. Evidence that exogenous E_2_ treatment enhances energy expenditure and improves systemic insulin sensitivity in OVX mice highlights its potential as a therapeutic approach for addressing these metabolic issues [161]. E_2_ exerts an insulin-sensitizing effect by improving glucose uptake, insulin signaling, and glucose transport in brain tissue [162]. The role of E_2_ in energy balance is mediated through ERα, and estrogen deficiency, along with the loss of ERα activity during menopause, contributes to metabolic disorders [163].

A particularly noteworthy finding is the connection between E_2_ deficiency and mitochondrial dysfunction. Mitochondria are essential for energy production and cellular homeostasis, and their dysfunction is implicated in various neuropsychiatric disorders, including depression. Estrogen’s influence on mitochondrial biogenesis, respiration, ATP production, and regulation of ROS may be crucial for understanding the metabolic basis of depression during menopause [164, 165]. Mitochondrial diseases have been observed in both depressed patients and animal models of depression [166, 167]. Additionally, the etiological mechanisms of depression, such as the production of inflammatory factors, reactive oxygen species, maintenance of synaptic plasticity, and neurotransmitter release, are also dependent on mitochondrial function and biogenesis [166, 168]. If mitochondrial dysfunction is indeed central to menopausal depression, interventions targeting mitochondrial health, such ai antioxidants and mitochondrial-targeted therapies, may offer promising solutions for improving both metabolic and mental health in perimenopausal women.

The complex interplay between E_2_ levels, metabolic regulation, mitochondrial function, and depression in perimenopausal women reveals important avenues for understanding the physical and mental health challenges of menopause. As estrogen levels fluctuate and eventually decline, they significantly affect energy metabolism, mitochondrial function, and the onset and progression of depression [163]. This dual impact underscores a profound connection between metabolic disturbances and mood disorders, a relationship that has been less explored in the literature compared to the independent investigation of these phenomena.

### 4.10 Gut Microbes

Recent studies have increasingly highlighted the close relationship between the gut microbiota and depression. Some researchers have proposed the “gut-brain axis” mechanism, suggesting that gut microbiota can influence brain function, thereby regulating emotions and behavior [169]. The impact of gut microbes on depression occurs through various pathways, including alterations in the glucose and amino acid metabolism, the HPA axis, neurotransmitter and short-chain fatty acid production, immune regulation, and neurogenesis [170-174]. Kelley *et al*. hypothesized a bidirectional relationship between depression and microbiota composition, which they tested through an experiment. Stool samples from individuals with depression exhibited a reduced number of species and lower phylogenetic diversity. Furthermore, the transplantation of faecal microbiota from depressed patients into microbiota-depleted rats induced depression-like behavioral and physiological changes in the recipient [175]. In addition, probiotics also have a positive effect on mood and depression [176, 177]. Gu *et al*. found that supplementation with the probiotic *Lactobacillus casei* alleviated depressive-like behaviors and reversed CUMS-induced alterations in gut microbiota structure [178]. In animal and clinical studies, lactobacillus and bifidobacteria species have been demonstrated to influence depressive and stress-related behaviors [176, 179].

In a recent study in which low estrogen caused significant changes in the intestinal flora, Fuhrman *et al*. discovered that postmenopausal women with a higher urinary ratio of hydroxylated estrogen metabolites to parent estrogen exhibited a more diverse gut microbiome [180]. A study of OVX animals revealed a higher ratio of *Firmicutes* to *Bacteroidetes* and *Escherichia coli* compared to the control group [181]. Similarly, E_2_ supplementation in male and OVX mice increased the relative abundance of *Bifidobacterium* and *Akkermansia* [182, 183]. This suggests that OVX alters the gut microbiome. Additionally, *Escherichia coli* infection weakens the host immune system, making mice more susceptible to estrogen deficiency, a condition reversed by estrogen treatment [184]. Moreover, Cross *et al*. reported that gut microbes are closely related to estrogen metabolism [185]. Experimental data indicates that female rats appear to be more resistant to intestinal damage and inflammatory responses than males. Gastrointestinal mucosal permeability in female rats fluctuates throughout the oestrous cycle [186]. Ovariectomy-induced estrogen deficiency impairs the intestinal mucosal barrier function [187]. The evidence supports that steroid hormones, in addition to their role in reproduction, are essential for maintaining gastrointestinal homeostasis and regulating disease susceptibility. Although estrogen influences the gut microbiome, the microbiome also substantially affects estrogen levels [181, 188]. Estrogen in the human body exists in two forms: bound and free. These forms can be interconverted through the involvement of intestinal microorganisms [189]. By secreting β-glucuronidase, the gut microbiome modulates estrogen levels. β-glucuronidase deconjugates estrogen, allowing it to bind to estrogen receptors and exert physiological effects. Only free, unbound estrogen is biologically active [190]. Sunmin *et al*. examined the effects of intracerebroventricular estrogen and progestin administration on menopausal symptoms in estrogen-deficient rats. They found that low doses of brain estrogen, and to a lesser extent progesterone, alleviated menopausal symptoms by reducing serum FSH levels and preserving gut microbiome diversity [191]. This suggests that estrogen and intestinal microbes are closely related, and their interaction may play a crucial role in the occurrence and development of PMD. The interaction between estrogen and gut microbiota indicates that hormones do not function in isolation but are part of a complex ecological system within the body. Fluctuations in estrogen during perimenopause and menopause, combined with changes in the gut microbiota, create a dynamic hormonal-ecological balance that may trigger or exacerbate mental health issues, such as depression. This could partly explain the high prevalence of depression during these stages. Estrogen's role in regulating gut microbiota diversity and its subsequent impact on brain function underscores the importance of considering the gut as a mediator in hormonal health. Furthermore, since gut microbes influence the bioavailability of estrogen through processes like β-glucuronidase-mediated deconjugation, the health of the gut microbiota may directly affect the body's response to estrogen therapy or other treatments aimed at alleviating menopausal symptoms.

### 4.11 Brain-gut Peptide

Brain-gut peptides, a crucial material basis for the function of the brain-gut axis, act as both neurotransmitters and hormones. They are primarily produced by intestinal, endocrine, and immune cells. These peptides are widely distributed throughout the central nervous system and the peripheral enteric nervous system [192]. Abnormal expression of brain-gut peptides mediates stress response, gastrointestinal motility disorders, appetite and feeding abnormalities, and gut microecological imbalance, which leads to depression, anxiety, gastrointestinal disorders, and metabolic diseases [193].

#### 4.11.1 Ghrelin

In 1999, Kojima *et al*. identified ghrelin, a novel brain-gut peptide composed of 28 amino acids, in gastric cells from rats [194]. Ghrelin plays a crucial role in stimulating the release of growth hormones, regulating food consumption, and maintaining energy balance. It also contributes to enhancing learning and memory functions, regulating reward-seeking behavior, and modulating anxiety and depression-like behavior [195-197].

Both rodents and humans provide substantial evidence supporting the antidepressant-like effects of ghrelin [198-200]. Notably, plasma ghrelin levels vary by gender, with women exhibiting higher circulating levels than men [201, 202]. Lower ghrelin levels were found in middle-aged women, whereas no differences are observed among younger and older men [203]. Therefore, estrogen may play a significant role in the regulation of ghrelin expression. Some studies suggest that estrogen replacement therapy, particularly oral estrogen therapy, increases active plasma ghrelin levels [204]. Additional animal research has shown that estrogen may directly influence the expression of growth hormone-releasing peptides. Fan *et al*. found that in ovariectomized mice, ghrelin generates antidepressant-like effects, potentially through ER involvement [205]. Estrogen treatment significantly stimulated ghrelin mRNA expression and production, an effect that was completely blocked by the ER antagonist ICI-182 780 [206]. Furthermore, co-localization of ghrelin and ERα was found in intact female rat gastric mucosal cells [207]. Future research should explore whether ghrelin-based interventions could offer a promising treatment for PMD. Moreover, understanding the synergistic relationship between ghrelin and other brain-gut peptides during hormonal transitions could open new avenues for managing not only depression but also comorbid metabolic disorders such as obesity and insulin resistance, which are prevalent in postmenopausal women.

#### 4.11.2 Neuropeptide Y (NPY)

NPY is a 36-amino-acid peptide that is widely distributed throughout the CNS [208]. It is associated with a range of biological functions, including food intake, cardiovascular regulation, circadian rhythms, seizure activity, and cognitive processes such as memory and learning [209, 210]. NPY levels are reduced in the limbic region of various depression models, including the Fawn Hooded and Flinders Sensitive Line rats, as well as rats with chronic mild stress [211, 212]. The NPY variant rs16139 and NPY Y2 receptor (Y2R) variant rs6857715 are associated with MDD. Postmortem examination of individuals who committed suicide and suffered from depression revealed diminished NPY-like immunoreactivity in the caudate nucleus and frontal cortex [213]. NPY exerts its antidepressant and anxiolytic effects by inhibiting the chronically overactive HPA axis in stress-related disorders [214].

NPY concentrations in the rat brain differ significantly before and after puberty, as well as in adult rats [215]. Research indicates that NPY expression is lower in females than in males in several regions of the brain, particularly when the brain is at rest or not under stress, which may impair their ability to cope with stress [216]. NPY is essential for central regulation of the gonadotropin axis, and its expression in the hypothalamus is influenced by estrogen. Estrogen increases the number of NPY neurons and NPY release in the hippocampus [217, 218]. A substantial abundance of NPY mRNA was found in the animals' arcuate nuclei during the pre-estrus or “low-estrogen” phase of the estrus cycle compared to the pre-estrus or “low-estrogen” phase [219]. Interestingly, males exhibit a greater number of NPY-containing cells in the arcuate nucleus than females, even during proestrus [220]. The Y1R in stress-related regions is regulated by estrogen. Hypothalamic Y1R expression increases during proestrus, the high-estrogen phase of the estrous cycle [221, 222], and estrogen response elements are present in the Y1R promoter. Estradiol regulates NPY expression in hippocampal formation interneurons in rats, and BDNF, an estrogen-regulated molecule, can also induce NPY expression [223].

*In vitro* studies on hypothalamic cell cultures have demonstrated that acute estrogen administration rapidly triggers a signal cascade essential for the long-term regulation of NPY gene expression [224]. In summary, these findings suggest that estrogen has both rapid and long-term effects on neuropeptide systems associated with mood and behavior. A key research gap in this area is understanding the temporal dynamics of NPY expression during different stages of the menstrual cycle and how these fluctuations correlate with mood. Furthermore, studies investigating the role of NPY in gender differences in stress resilience may inform the development of personalized therapies. NPY-based pharmacological interventions could serve as adjunctive treatments for PMD, especially for individuals whose depressive symptoms are exacerbated by stress.

#### 4.11.3 Arginine-vasopressin (AVP)

AVP, a nonapeptide posterior hormone of the pituitary, is primarily synthesized and secreted in the hypothalamic PVN and supraoptic nucleus (SON) [225]. AVP is essential for regulating water balance and blood pressure; it also plays a crucial role in modulating emotions and social behaviors, including stress, anxiety, and depression. Gerben *et al*. found that the expression of AVP mRNA was significantly elevated in both SON and PVN in depressed patients compared to controls [226]. In 1996, Purba *et al*. reported that depressed patients exhibited a higher number of neurons expressing the V1b vasopressin receptor subtype than healthy controls in a post-mortem analysis [227].

Estrogen exposure can account for gender differences in AVP levels, as AVP is regulated by estradiol [228]. Estradiol regulates the activity of hypothalamic large cell neurons and modulates AVP release, expression, and immunoreactivity in the SON and PVN. Recent studies have shown that ERs are expressed in the magnocellular AVP neurons of the SON and PVN [229-231]. Acute E_2_ injection in ovariectomized rats significantly reduced the number of AVP immunoreactive neurons in the SON and PVN [229]. Studies conducted *in vitro* on ER-transfected cells have demonstrated that activation of ERβ by estradiol inhibits the AVP promoter activity [232]. Nomura *et al*. further showed that the downregulation of AVP mRNA and protein in PVN was abolished in betaERKO mice [228]. AVP neurons are primarily regulated by estrogen *via* ERβ, as evidenced by gene and protein expression in rats and knockout mice [233]. However, the possibility of estrogen acting indirectly on AVP neurons *via* ERα in rats cannot be ruled out [234]. There are still several unresolved questions regarding the precise mechanisms by which estrogen influences AVP expression, particularly concerning gender differences. Further research is needed to determine whether AVP regulation is specific to estrogen-induced changes in the hypothalamus or whether it involves broader interactions with other neuroendocrine systems.

## 5 THERAPEUTIC POTENTIAL OF PHYTOESTROGENS IN PERIMENOPAUSAL DEPRESSION

Phytoestrogens are naturally occurring compounds present in various plants [235, 236]. These compounds share structural similarities with estrogen, allowing them to bind to estrogen receptors in the body and exert estrogen-like effects [237]. The main types of phytoestrogens include: (1) Isoflavones, which are found in soybeans, tofu, fermented soy products, and other legumes; (2) Lignans, primarily found in seeds (especially flaxseeds), whole grains, and certain fruits and vegetables; and (3) Coumestans, which are predominantly found in sprouts, particularly alfalfa and clover [237, 238].

Several clinical studies have confirmed the potential of phytoestrogens in alleviating perimenopausal depression [239, 240]. For instance, soy isoflavones (such as genistein and daidzein) have been shown to effectively reduce depressive symptoms in postmenopausal women [241]. A randomized controlled trial demonstrated that women who took soy isoflavones experienced significant improvements in mood [242]. Similarly, flaxseeds, which are rich in lignans, have been shown to alleviate anxiety and depression symptoms in perimenopausal women [243]. The mechanism of action of phytoestrogens primarily involves binding to estrogen receptors (ERα and ERβ) in the brain. These receptors are widely distributed in regions associated with mood regulation, such as the hippocampus, hypothalamus, and cortex. By binding to these receptors, phytoestrogens can enhance serotonin activity, improve neuroplasticity, regulate the HPA axis, and reduce oxidative stress [244-246].

Phytoestrogens offer a promising alternative treatment for perimenopausal depression, particularly for women who prefer natural therapies. Compared to traditional synthetic hormones, phytoestrogens have fewer side effects and can be used long-term without triggering common adverse reactions associated with estrogen replacement therapy [247]. Additionally, phytoestrogens are linked to improved cardiovascular health, enhanced bone density, and a reduced risk of breast and prostate cancer. The effects of phytoestrogens vary among individuals, influenced by factors such as genetic differences, gut microbiota composition, and the source of phytoestrogens. While some women may experience significant effects, others may observe only mild benefits. Although generally considered safe, phytoestrogens may affect hormone-sensitive conditions, such as breast and ovarian cancer [248]. Standardization of phytoestrogen supplement dosages remains a challenge, which may contribute to variations in effectiveness across different studies and practical applications. Therefore, women considering phytoestrogens as a treatment option should collaborate with their healthcare providers to assess their individual health conditions and integrate lifestyle interventions such as diet, exercise, and stress management.

## CONCLUSION

This study systematically investigates the pathogenesis of perimenopausal depression from multiple perspectives, focusing on the roles of estrogen receptors, neurotransmitters, inflammatory responses, the neuroendocrine system, neuroplasticity, oxidative stress, energy metabolism, mitochondrial function, and the microbiome-gut-brain axis. Specifically, estrogen receptors in the brain not only influence the synthesis and release of neurotransmitters but also significantly affect mood and cognitive functions through the regulation of neuroendocrine function, neuroplasticity, and antioxidant mechanisms. Moreover, mitochondrial dysfunction and energy metabolism abnormalities are critical factors in this process, exacerbating the onset and progression of perimenopausal depression. The relationship between the gut-brain axis and mood disorders provides a novel perspective, although understanding of this mechanism remains in its early stages. The complexity of the gut-brain axis is manifested not only in the diversity and functions of the microbiota but also in the signaling pathways involving brain-gut peptides, immune responses, and neuroendocrine interactions. Fig. (**4**) illustrates the intricate relationships between these biological mechanisms, emphasizing the multidimensional and multifactorial nature of perimenopausal depression. Notably, perimenopausal depression is not triggered by a single factor but results from the interaction of various biological mechanisms. For example, estrogen regulation of the neuroendocrine system depends not only on its direct effects on neurotransmitters but may also indirectly influence mood and depressive symptoms through its effects on the immune system, oxidative stress responses, and alterations in the gut microbiota. Future research should prioritize examining the dynamic interactions between these factors to identify potential common pathways or regulatory nodes.

Estrogen replacement therapy holds substantial potential for alleviating perimenopausal depression. Studies indicate that appropriate estrogen replacement therapy can help mitigate mood disorders caused by estrogen deficiency. However, this therapy carries certain risks, particularly with long-term use, which may increase the incidence of breast cancer, cardiovascular diseases, and other health conditions. Therefore, future clinical studies should focus on personalized estrogen replacement therapy regimens, assess their effectiveness and risks in various populations, and explore their combined use with other therapeutic methods. Phytoestrogens, considered a safer alternative due to their lower hormonal side effects, are a potential treatment option. However, the efficacy and safety of phytoestrogens vary significantly between individuals, and some studies suggest their effects on alleviating depressive symptoms are less pronounced than those of synthetic estrogens. Consequently, the therapeutic effects of phytoestrogens require further clinical validation.

In summary, future research should place greater emphasis on interdisciplinary integration, utilizing longitudinal and multidimensional research methods and examining the interactions between biological mechanisms and individual differences. Exploring personalized treatments and the potential role of the gut-brain axis in perimenopausal depression may open new avenues for therapy. Only through in-depth mechanistic studies and the accumulation of clinical data can we provide stronger support for the early diagnosis, precise treatment, and long-term management of perimenopausal depression.

## AUTHORS’ CONTRIBUTIONS

The authors confirm their contribution to the paper as follows: YQ. L. Wrote the first draft. W.Y. Made major revisions to the logic of this article. XY. F., BY. G., and RJ. C. Participated in the revision of the manuscript. All authors reviewed the results and approved the final version of the manuscript.

## Figures and Tables

**Fig. (4):**
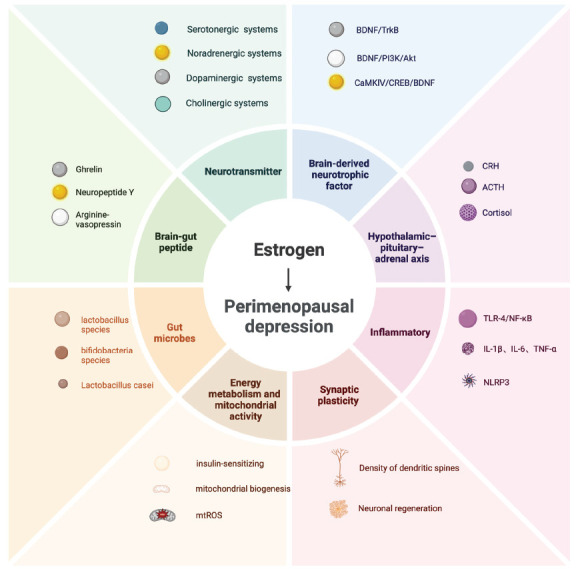
The mechanism of estrogen against perimenopausal depression. **Abbreviations**: BDNF, brain-derived neurotrophic factor; TrkB, Tyrosine receptor kinase B; PI3K, phosphatidyl inositol 3-kinase; AKT, serine/threonine protein kinase; CREB, cAMP response element-binding protein; CaMK IV, calmodulin-dependent protein kinase IV; TLR4, Toll-like receptor 4; NF-κB, nuclear factor κB; TNF-α, Tumor necrosis factor-α; IL, Interleukin; NLRP3: nucleotide-binding and oligomerization domain-like receptor family pyrin domain-containing 3; CRH, corticotropin-releasing hormone; ACTH, adrenocorticotrophic hormone.

## LIST OF ABBREVIATIONS

ACTH: Adrenocorticotrophic Hormone
AVP: Arginine-vasopressin
BDNF: Brain-derived Neurotrophic Factor
CaMK IV: Calmodulin-dependent Protein Kinase IV
CREB: cAMP Response Element-binding Protein
CRH: Corticotropin-releasing Hormone
CUMS: Chronic Unpredictable Mild Stress
DA: Dopamine
DRN: Dorsal Raphe Nucleus
E2: Estradiol
ERs: Estrogen receptors
ET: Estrogen Therapy
FSH: Follicle-stimulating Hormone
GABA: γ-aminobutyric Acid
GC: Glucocorticoid
GR: Glucocorticoid Receptor
HPA: Hypothalamic-pituitary-adrenal
5-HT: 5-hydroxytryptamine
IL-1β: Interleukin-1β
LPS: Lipopolysaccharide
MAO-A: Monoamine Oxidase A
MAPK: Mitogen-activated Protein Kinases
MDD: Major Depressive Disorder
NF-κB: Nuclear Factor κB
NLRP3: Nucleotide-binding and Oligomerization Domain-like Receptor Family Pyrin Domain-Containing 3
NPY: Neuropeptide Y
OVX: Ovariectomy
PI3K: Phosphatidylinositol 3-kinase
PLC: Phospholipase C
PMD: Perimenopausal Depression
PVN: Paraventricular Nucleus
ROS: Reactive Oxygen Species
SOD2: Superoxide Dismutase
STRAW: Stages of Reproductive Aging Workshop
TLR-4: Toll-like Receptor 4
TNF-α: Tumour Necrosis Factor-α
TrkB: Tyrosine Receptor Kinase B
